# Effects of **Achillea wilhelmsii** on rat’s gastric acid output at basal, vagotomized, and vagal-stimulated conditions

**DOI:** 10.4103/0973-1296.71791

**Published:** 2010

**Authors:** S. Niazmand, E. Khooshnood, M. Derakhshan

**Affiliations:** *Department of Physiology, Medical School, Mashhad University of Medical Sciences, Mashhad, Iran*; 1 *Department of Biology, Sciences School, Azad University of Mashhad, Mashhad, Iran*; 2 *Department of Clinical Microbiology and Virology in Ghaem Hospital and Buali Research Institute, Mashhad University of Medical Sciences, Mashhad, Iran*

**Keywords:** Achillea wilhelmsii, gastric acid, vagotomy, vagus nerve

## Abstract

**Background:**

**Achillea** is a plant widely used in traditional medicine for gastrointestinal disorders. There are some reports on gastrointestinal effects of **Achillea**, such as antiulcer, antibacterial, hepatoprotective, choleretic, and antispasmodic. To investigate the effects of aqueous–ethanol extract of **Achillea wilhelmsii** on rat’s gastric acid output in basal, vagotomized (VX), and vagal-stimulated conditions.

**Materials and Methods:**

24 male Wistar rats were randomly divided into 2 groups: control and test. Gastroduodenostomy was performed for each rat. Gastric content was collected for 30 min by washout technique. One milliliter of 3 doses (0.5, 1, and 2 mg/kg) was introduced into the stomach of each rat in the test group and the same volume of saline was used in the control group. Total titratable acid was measured by a titrator.

**Results:**

The extract inhibited acid output significantly in basal condition by 1 and 2 mg/kg doses (*P* < 0.05) but in VX condition this inhibitory effect on acid output disappeared and the 1 and 2 mg/kg doses increased acid output significantly (*P* < 0.05 and *P* < 0.001, respectively). The extract showed a reduction in the acid output in vagal-stimulated condition by 1 and 2 mg/kg doses, which were not statistically significant.

**Conclusion:**

These results showed an inhibitory effect of *A. wilhelmsii* extract on acid output in basal condition. The inhibitory effect of the extract was exerted via gastric vagal parasympathetic nerve.

## INTRODUCTION

Gastrointestinal disorders due to abnormal metabolic or physical processes, such as gastric and duodenal ulcers, gastritis, dyspepsia, hyperchlorhydria, or functional gastrointestinal disorders, such as irritable bowel syndrome (IBS), are highly prevalent worldwide.[1] Several metabolic and physical disorders related to gastric acid and gastric motility disturbances have been treated by using synthetic and herbal medicines. Medicinal plants have been used for over 2000 years and an increasing attention has been paid to herbal medicine products because of their effectiveness and lower cost in recent years. **Achillea** is one of the most important genera of the Compositae family and comprises more than 120 spices. Several effects, such as anti-inflammatory,[2] antibacterial,[3,4] antihypertensive, and anti-hyperlipidemia,[5] and antitumor,[6,7] have been reported for **Achillea**. It is widely used in traditional medicine for gastrointestinal disorders[8] and there are some reports of its effects, such as antispasmodic,[9,10] choleretic,[11] antiulcer,[12] antibacterial (*Helicobacter pylori*),[13] and hepatoprotective, on the gastrointestinal tract.[10] **Achillea wilhelmsii** is the major spice, which is grown in Iran and widely used in Iranian traditional medicine for gastrointestinal disorders. It has chemical components, including flavonoids, alkaloids (achilleine), cineol, borneol, α- and β-pinen, camphor, caryophyllene, thujene, rutin, sesquiterpenoids, and monoterpenoids.[14–17] No reports on its effect on gastric acid output are available at the moment. Thus, the aim of this study was to investigate possible effects of aqueous-ethanol extract of *A. wilhelmsii* on gastric acid output. To further clarify the interaction of the extract with gastric vagal parasympathetic system, we considered 3 conditions: basal, vagotomized, and vagal stimulated.

## Materials and Methods

### Plant material

The aerial part of *A. wilhelsii* was collected from South Khorasan Province, Iran, and identified by botanists in the Herbarium of Pharmacy School, Mashhad University of Medical Sciences (voucher No. 142-2012-4) and then dried at room temperature.

### Extraction method

Three hundred grams of aerial parts of the plant were soaked in ethanol (50%) for 24 h and paper filter was used to filter the solute after mixing. The solution was then dried using a 40°C oven for 36 h. The dried extract was dissolved in the distilled water to make 0.5, 1, and 2 mg/kg doses.

### Experimental procedure

The experiment was conducted using 24 male Wistar rats (weighed 200–250 g). The animals were kept in a 20°C±2°C temperature with a 12 h light/dark cycle and fed with standard diet and tap drinking water. The study was permitted by the Institutional Animal Ethics Committee of Mashhad University of Medical Sciences (MUMS). Animals were randomly divided into 2 groups: control and test. The surgical procedure was performed for all the groups. In the test group, the extract was administered and in the control group, saline was used instead of the extract. All the rats were not fed for 24 h but were allowed access to drinking water before operation. To avoid the effect of circadian rhythm, the experiments were started at 8:00 AM every day. Anesthesia was induced by sodium thiopental (50 mg/kg; ip) and cervical esophagus was ligated after tracheostomy. A midline incision was performed on abdomen and a silicone tube (2.5 mm in external diameter) was introduced to the stomach via duodenum.[18] For emptying the remaining gastric contents, the stomach was washed with 1 mL of 37°C normal saline (pH = 7). In VX condition, vagus nerve in the cervical region was dissected and the extract was introduced to the stomach. In vagal-stimulated condition, the vagus nerve was stimulated (15 V, 4 Hz, width 0.05 ms, 30 min) by a stimulator (Harvard Apparatus, England).[19] Gastric acid output was measured 30 min after the extract was introduced to the stomach and the acid output was calculated and reported as mEq/30 min. The gastric content was titrated by NaOH (0.01 N). To measure the gastric acid output in the test group, 1 mL of 0.5, 1, and 2 mg/kg of the aqueous–ethanol extract was introduced into the stomach of each rat for 30 min.

### Data analysis

The data were represented as mean ± SEM and analyzed by Wilcoxon signed rank test. *P* < 0.05 was considered significant.

## RESULTS

**Figure 1:**
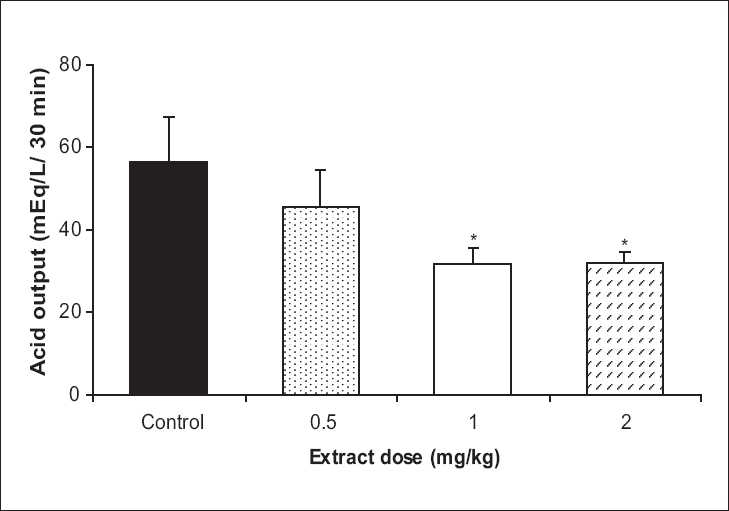
The effect of **Achillea wilhelmsii** extract on gastric acid output at basal condition. The gastric acid output significantly decreased with 1 and 2 mg/kg doses of the extract in comparison to the control group (n = 12, *P < 0.05)

**Figure 2:**
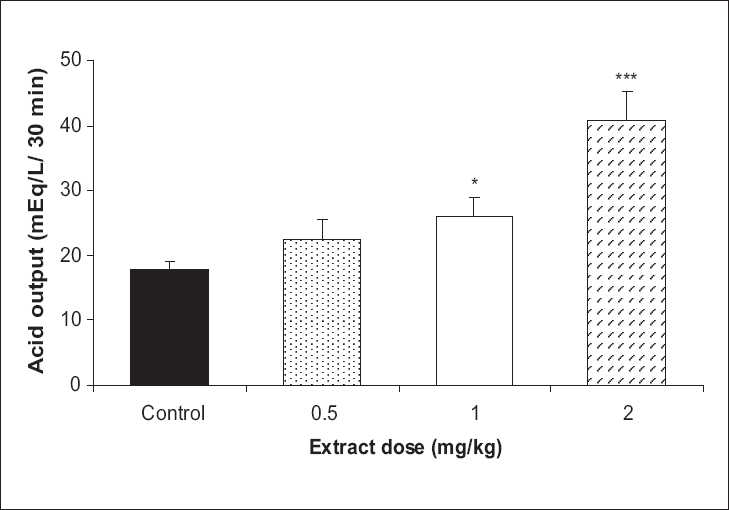
The effect of **Achillea wilhelmsii** extract on gastric acid output at vagotomized condition. The gastric acid output significantly increased with 1 and 2 mg/kg doses of the extract in comparison to the control group (n = 12, *P < 0.05, ***P < 0.001)

**Figure 3:**
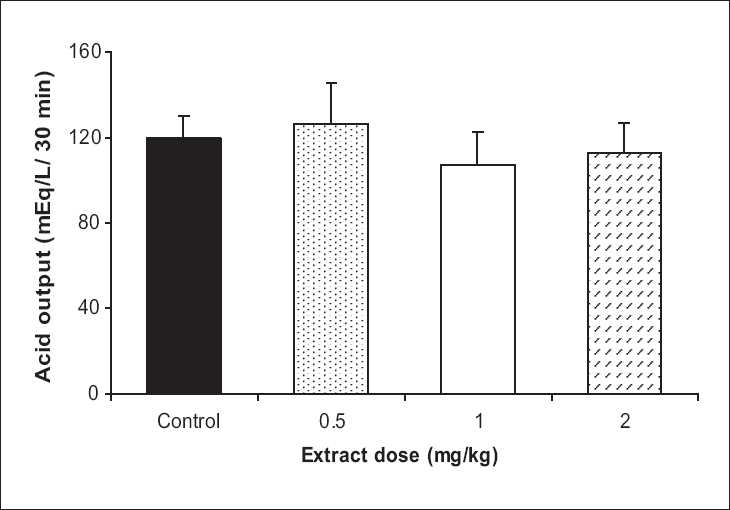
The effect of **Achillea wilhelmsii** extract on gastric acid output at vagal stimulation condition. The extract showed a reduction in gastric acid output by 1 and 2 mg/kg doses, which statistically were not significant in comparison to the control group (n = 12)

Basal acid output in the test group showed a significant decrease with 1 and 2 mg/kg doses of the extract in comparison to the control group (45.2% and 43.2%, respectively, *P* < 0.05) [Figure 1]. In VX condition, the test group showed a rise in the acid output, which was significant by the 1 and 2 mg/kg doses in comparison to the control group (45% and 129%, *P* < 0.05 and *P* <0.001, respectively) [Figure 2]. In vagal stimulation condition, the extract showed a reduction in the acid output by 1 and 2 mg/kg doses (10% and 7%, respectively), which were not statistically significant in comparison to the control group [Figure 3].

## DISCUSSION

Recent studies have revealed some effects of **Achillea** on gastrointestinal tract as mentioned in the Introduction, but previous studies have not precisely clarified the probable mechanism involved in gastric acid output. The results of this study indicate that the aqueous–ethanol extract of *A. wilhelmsii* had an inhibitory effect on gastric acid output in basal condition. This inhibitory effect disappeared at VX condition and prominent excitatory effects were shown; thus the inhibitory effect of the extract in basal condition should be exerted via gastric vagal parasympathetic nerve. The doses of 1 and 2 mg/kg showed inhibitory effect on gastric acid output in basal condition, but at VX condition they had excitatory effect on gastric acid output in comparison with the control group. This may indicate the presence of compound/s with an excitatory effect on gastric acid secretion in the extract that act through processes other than gastric parasympathetic system. The inhibitory effect of the extract on gastric acid output in vagal-stimulated condition was not significant, thus it may be concluded that the extract was unable to overcome the excitatory effect of the vagal stimulation on gastric acid secretion. The previous studies showed that Achillea reduced the gastric acidity and increase the mucus synthesis and excretory function of the stomach.[20,21] The antiulcer and gastroprotective effect of **Achillea** may be partly due to the reductive potential of gastric acid output at basal condition. There is another study that shows cineole, one of the main constituent of *A. wilhelmsii*, has inhibitory effect on gastric acid secretion that leads to its gastroprotective effect.[22]

## CONCLUSION

Our findings show that the *A. wilhelmsii* extract has an inhibitory effect on gastric acid output in basal condition via the gastric parasympathetic nerve. But the extract has no effect in vagal-stimulated condition.
